# Daily Treatment Monitoring for Patients Receiving Home-Based Peritoneal Dialysis and Prediction of Heart Failure Risk: mHealth Tool Development and Modeling Study

**DOI:** 10.2196/56254

**Published:** 2025-03-03

**Authors:** Jia Wu, Youjia Zeng, Jun Yang, Yutong Yao, Xiuling Xu, Gaofeng Song, Wuyong Yi, Taifen Wang, Yihou Zheng, Zhongwei Jia, Xiangyu Yan

**Affiliations:** 1 Shenzhen Hospital of Traditional Chinese Medicine Shenzen China; 2 Department of Global Health School of Public Health Peking University Beijing China; 3 School of Disaster and Emergency Medicine Tianjin University Tianjin China; 4 Key Laboratory of Medical Rescue Key Technology and Equipment Ministry of Emergency Management Tianjin China

**Keywords:** peritoneal dialysis, mHealth, patient management, heart failure, prediction model

## Abstract

**Background:**

Peritoneal dialysis is one of the major renal replacement modalities for patients with end-stage renal disease. Heart failure is a common adverse event among patients who undergo peritoneal dialysis treatment, especially for those who undergo continuous ambulatory peritoneal dialysis at home, because of the lack of professional input-output volume monitoring and management during treatment.

**Objective:**

This study aims to develop novel mobile health (mHealth) tools to improve the quality of home-based continuous ambulatory peritoneal dialysis treatment and to build a prediction model of heart failure based on the system’s daily treatment monitoring data.

**Methods:**

The mHealth tools with a 4-layer system were designed and developed using Spring Boot, MyBatis Plus, MySQL, and Redis as backend technology stack, and Vue, Element User Interface, and WeChat Mini Program as front-end technology stack. Patients were recruited to use the tool during daily peritoneal dialysis treatment from January 1, 2017, to April 20, 2023. Logistic regression models based on real-time treatment monitoring data were used for heart failure prediction. The sensitivity, specificity, accuracy, and Youden index were calculated to evaluate the performance of the prediction model. In the sensitivity analysis, the ratio of patients with and without heart failure was set to 1:4 and 1:10, respectively, to better evaluate the stability of the prediction model.

**Results:**

A WeChat Mini Program named Futou Bao for patients and a patient data management platform for doctors was developed. Futou Bao included an intelligent data upload function module and an auxiliary function module. The doctor’s data management platform consisted of 4 function modules, that is, patient management, data visualization and marking, data statistics, and system management. During the study period, the records of 6635 patients who received peritoneal dialysis treatment were uploaded in Futou Bao, with 0.71% (47/6635) of them experiencing heart failure. The prediction model that included sex, age, and diastolic blood pressure was considered as the optimal model, wherein the sensitivity, specificity, accuracy, and Youden index were 0.75, 0.91, 0.89, and 0.66, respectively, with an area under the curve value of 0.879 (95% CI 0.772-0.986) using the validation dataset. The sensitivity analysis showed stable results.

**Conclusions:**

This study provides a new home-based peritoneal dialysis management paradigm that enables the daily monitoring and early warning of heart failure risk. This novel paradigm is of great value for improving the efficiency, security, and personalization of peritoneal dialysis.

## Introduction

Peritoneal dialysis is one of the 3 major renal replacement modalities (ie, hemodialysis, peritoneal dialysis, and kidney transplantation) for end-stage renal disease (ESRD) [1-3]. Continuous ambulatory peritoneal dialysis (CAPD) gradually becomes the mainstream mode for peritoneal dialysis [4]. Patients can receive the treatment at home because the treatment site environment needs less treatment resources and is flexible [5,6]. Compared with hemodialysis, peritoneal dialysis as a home-based, cost-saving renal replacement therapy has several advantages, including protection of residual kidney function, stable hemodynamics, and less interference with normal family, work, and social life. Peritoneal dialysis therapy has been recognized and accepted by patients with ESRD.

One common and serious adverse event in patients undergoing CAPD is capacity-overloaded heart failure. Previous studies have shown a high prevalence of congestive heart failure among patients who receive peritoneal dialysis treatment. A multicenter cohort study in mainland China conducted in 2012 reported that heart failure occurred in 47.3% of the patients who underwent peritoneal dialysis [7]. Contemporaneously, Wang et al’s [8] study conducted in Hong Kong showed a heart failure prevalence of 39% in patients treated with peritoneal dialysis. Although the prevalence reported by subsequent studies showed a decrease to 29% in Wang et al’s study [9] and to 19.6% in Sun et al’s study [10], the prevalence remained at a high level. Therefore, the prevention and early detection of heart failure in patients undergoing peritoneal dialysis treatment is a vital issue that cannot be ignored.

Although operating peritoneal dialysis treatment at home has many advantages, there are still several treatment bottlenecks in the traditional home-based peritoneal dialysis approach. First, because patients manage the treatment at home, doctors cannot provide timely and accurate follow-up. Second, the effectiveness of peritoneal dialysis, the occurrence of adverse events represented by heart failure, and the quality of life of patients are closely related to the training and management level of the medical institutions and the self-management levels of the patients. Third, it is difficult to implement good remote whole-process quality management of patients and prevent adverse events in a timely and effective manner for patients receiving CAPD at home.

Given the treatment bottleneck in the traditional home-based peritoneal dialysis method, it is essential to develop a user-friendly remote intelligent treatment management platform. The internet, big data, and other mobile health (mHealth) technologies can enable seamless integration of mobile equipment, systems, and home-based CAPD treatment operations to conduct timely detection and early intervention of adverse events represented by heart failure through analyzing the automatically collected data by the system.

The aim of this study was to develop mHealth tools and systems to improve the efficiency, accuracy, and personalization of the home-based peritoneal dialysis treatment and to build a prediction model of heart failure in patients with peritoneal dialysis based on the systems’ daily treatment monitoring data.

## Methods

### mHealth Tool Design and Development Procedures

The mHealth tools developed in this study were intended to promote the intelligence of the home peritoneal dialysis treatment in the following 4 aspects.

1. Automated data collection: Through the Bluetooth connection device, the system enables the automatic collection of peritoneal dialysis data, including peritoneal dialysis solution weight and patient’s weight data, which can reduce manual operation and the possibility of data input error.
2. Daily data analysis: The system timely processes and analyzes the uploaded data and provides intuitive data visualization to help doctors better understand the patient’s treatment status.
3. Remote treatment monitoring: In order to improve the efficiency and convenience of treatment, patients do not need to go to the hospital frequently for treatment, and doctors can remotely monitor and manage the treatment process of patients.
4. Personalized treatment plan: Through the real-time data analysis and treatment monitoring, doctors can develop personalized treatment plans according to the specific conditions of patients and improve the treatment effect.

**Figure 1:**
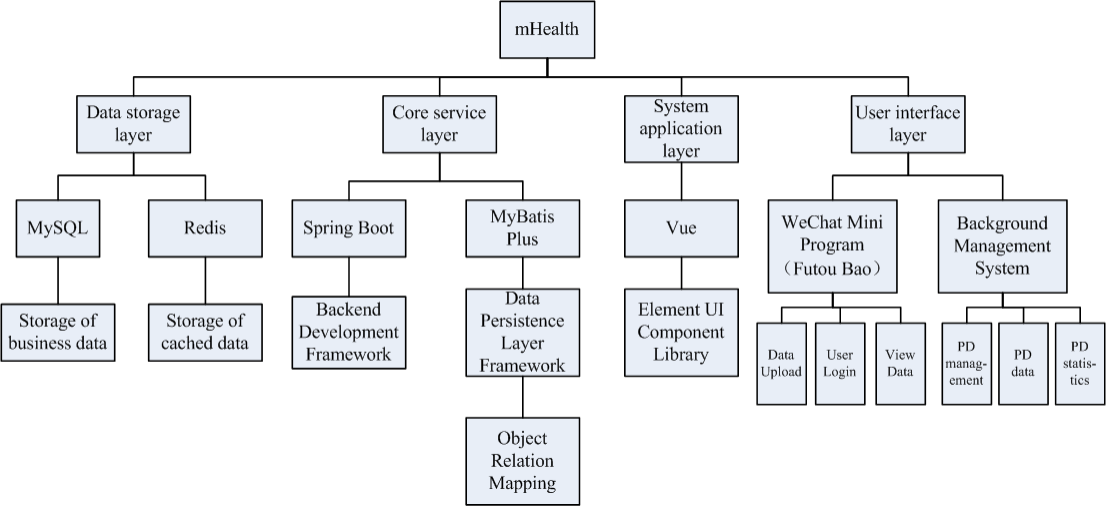
Architecture of the mobile health system. mHealth: mobile health; PD: peritoneal dialysis; UI: user interface.

In order to improve the mHealth system’s usage convenience, this study plans to use Spring Boot, MyBatis Plus, MySQL, and Redis as backend technology stack, and Vue, Element User Interface, and WeChat Mini Program as the front-end technology stack. The whole system is divided into 4 layers: data storage layer, core service layer, system application layer, and user interface layer. This architecture could make the system efficient, reliable, and extensible, and meet the functional and performance requirements of peritoneal dialysis treatment management. The details of each layer are as follows (Figure 1):

1. Data storage layer: This layer is responsible for data storage and management. MySQL database is used to store various data, including user information and medical records. Redis is used as a nonrelational database to store login token and treatment time point information as a cache to improve the ability to query data.
2. Core service layer: This layer is the core operation processing layer of the system, responsible for realizing the core functions and business logic of the system. Spring Boot framework was adopted as the backend development framework, combined with MyBatis Plus as the persistence framework. Each function module of the system was developed through the annotation method of Spring Boot and used the Object Relational Mapping provided by MyBatis Plus to interact with the MySQL database.
3. System application layer: This layer is responsible for receiving and handling user requests. A single-page application based on Vue framework was used as the front-end technology of the background management system, and Element User Interface was used as the user interface component library to build the web pages and interactive interface of the system. For patient users, the development framework of the WeChat Mini Program was adopted to realize the user’s access and use of the system in WeChat (the most popular mobile app in China).
4. User interface layer: This is the interface layer for the system to interact with users and is responsible for displaying the data and functions of the system to users in an intuitive and friendly way.

### Design of the Longitudinal Study and Model Prediction

From January 1, 2017, to April 20, 2023, a longitudinal study was conducted based on the usage of the Futou Bao system among outpatients of Shenzhen Hospital of Traditional Chinese Medicine. Patients who met the following criteria were recruited and included in the longitudinal study. The inclusion criteria for the participants were as follows.

1. Met the clinical standard of home peritoneal dialysis, including (1) patients with ESRD who needed continuous renal replacement therapy and chose peritoneal dialysis as the kidney replacement therapy; (2) patients underwent peritoneal dialysis catheterization, and the peritoneal dialysis catheter was inserted, which met the requirements of the peritoneal dialysis treatment; and (3) patients accepted the training of home peritoneal dialysis operation and obtained the qualification of self-completion of peritoneal dialysis operation after the training.
2. Intended to receive medical follow-up in Shenzhen Hospital of Traditional Chinese Medicine.
3. Had no difficulty in using smartphones and WeChat.
4. Willing to follow the procedure of the mHealth tool for operate peritoneal dialysis at home and sign the informed consent document.

The exclusion criteria included (1) patients diagnosed with heart failure and met either of the following 2 conditions, namely, acute heart failure classified as New York Heart Association class III or above and nonacute heart failure classified as New York Heart Association class II or below but combining with peripheral edema; and (2) patients had other serious physical and mental diseases that they could not complete the peritoneal dialysis at home with the help of the mHealth tool that we developed. In addition, in the process of using the mHealth tool, when clinical evaluation indicated that the patient did not meet the conditions of home peritoneal dialysis and needed hospitalization, the use of the mHealth tool would be stopped immediately.

Prior to patient recruitment, a specialized patient home peritoneal dialysis management team was selected, including professional doctors and nurses from the Department of Nephrology. Training for the patient management team was conducted, including home peritoneal dialysis operation and system use methods, patient recruitment methods, patient follow-up methods, and management methods. Thereafter, the above management team recruited the patients in their daily work and conducted one-on-one training for the patients who met the enrollment criteria. Patients were asked to use the mHealth tool during each peritoneal dialysis at home. Designated doctors and nurses were assigned to patients for treatment monitoring and follow-up.

During the usage of Futou Bao, patients’ daily peritoneal dialysis treatment data were recorded in the system, which were obtained to create the dataset of the modeling process. In the dataset, identifiable information of the patients, such as names, were replaced with a string of encrypted ID numbers to blind the data analysts during analysis. Sex, age, time duration of peritoneal dialysis, weight, urine volume, diastolic blood pressure, systolic blood pressure, and pulse pressure difference were obtained and included in the dataset as prediction variables to predict the onset of heart failure. Because the dialysate recommended in the home-based peritoneal dialysis was 1.5% glucose concentrations, the type of dialysate was not considered as a prediction variable in this study. For the heart failure outcome, because the occurrence of heart failure was always a progressive process among patients who received peritoneal dialysis, the 15 days before the date of hospitalization for heart failure was defined as the time duration of progression of heart failure. The data collected in that time duration were considered to be related to heart failure. Due to the careful and close patient management, the exact time of heart failure hospitalization could be obtained so as to avoid information bias. Because heart failure occurred infrequently, we randomly selected 6 times the number of patients with heart failure from the data of patients without heart failure to form the study dataset (number of patients with heart failure vs number of patients without heart failure = 1:6). After that, 80% of the data in the study dataset were randomly selected as the training dataset, while 20% were selected as the validation dataset of the prediction model. The sampling method without replacement was adopted in the above process. In the sensitivity analysis, we set the ratio of patients with and without heart failure to 1:4 and 1:10, respectively, to better evaluate the stability of the prediction model.

### Statistical Analysis

The therapeutic effect and process of patients undergoing peritoneal dialysis treatment is self-specific, which should be considered in conjunction with the patient’s own treatment monitoring data. For improving the adaptability of prediction model, it is more important and valuable to observe patients’ own indicator value changes, including weight, urine volume, and blood pressure. Considering the different baseline data of each patient, the least squares method was used to fit the data of each patient, and discrete points were defined as the actual data that were not in the confidence intervals of the fitted data calculated by least squares method [11]. With the help of this method, the urine volume, diastolic blood pressure, systolic blood pressure, and pulse pressure difference were converted from numerical variables to categorical variables (ie, normal, abnormal). In this study, both α=.05 and α=.01 were used as the CI criterion (ie, 95% CI and 99% CI) to divide the categories of variables, so as to choose the optimal model.

**Figure 2:**
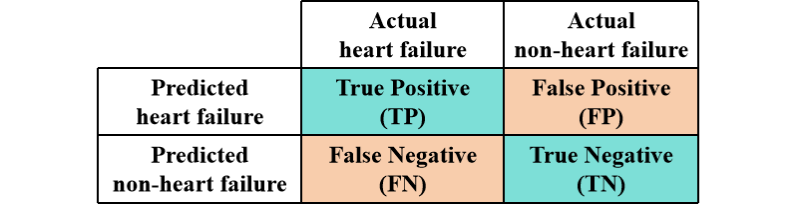
Definitions of true positive, false positive, false negative, and true negative.

Logistic regression models were used as prediction models. Four models were built using the training dataset. Models 1 and 2 used the variables based on the 95% CI criterion, while models 3 and 4 used the variables based on the 99% CI criterion. In models 1 and 3, all of the variables were included, while models 2 and 4 used the backward method and only included variables that were statistically significant. Receiver operating characteristic curves were presented, and the corresponding area under the curve (AUC) values were calculated. The AUC value and Akaike’s information criterion (AIC) value were considered comprehensively to select the optimal model, which showed the highest AUC value and the lowest AIC value. Using the validation dataset, the sensitivity, specificity, accuracy, and Youden index of the optimal model were calculated, and the receiver operating characteristic curve and decision curve were presented to evaluate the performance of the prediction model comprehensively. The specific calculation formulas were as follows (Figure 2):

Sensitivity = True Positive / (True Positive + False Negative) (Formula 1)

Specificity = True Negative / (False Positive + True Negative) (Formula 2)

Accuracy = (True Positive + True Negative) / (True Positive + False Positive + False Negative + True Negative) (Formula 3)

Youden index = (Sensitivity + Specificity) – 1 (Formula 4)

A 2-sided *P* value of .05 was used for null hypothesis tests. Statistical analyses were conducted using SPSS version 19.0 (IBM Corp) and R version 4.3.0 (R Core Team).

### Ethics Approval

This study was approved by the medical ethics committee of Shenzhen Hospital of Traditional Chinese Medicine (B2019043 and K2021-036-01). Written informed consent was obtained from the participants. In the analysis process, identifiable information of the patients (ie, names) was replaced with a string of encrypted ID numbers to protect patients’ privacy. There was no compensation (money or gift) provided to the participants during the implementation of the study. However, participants received careful and fair health services and management of home-based peritoneal dialysis treatment.

## Results

### mHealth Tools Development: Modules and Functions

**Figure 3:**
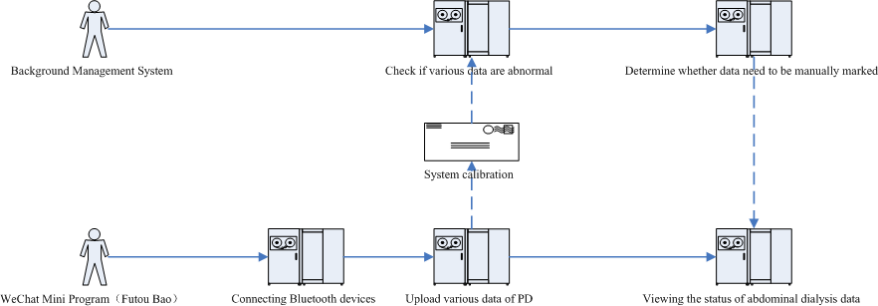
Functional flow diagram. PD: peritoneal dialysis.

In order to realize safe and convenient peritoneal dialysis for patients at home and to make it convenient for doctors to know the treatment and the physical condition of the patients in real time, an mHealth system was developed, comprising a WeChat Mini Program for patients named Futou Bao (“腹透宝” in Chinese) and a patient data management platform used by doctors (Figure 3).

### WeChat Mini Program (Futou Bao) for Patients

Futou Bao is a WeChat Mini Program that patients can use easily on WeChat, which is one of the most popular mobile social apps in China by scanning the quick response code or searching the name (“腹透宝” in Chinese) through WeChat. The main function of Futou Bao is the intelligent data upload function for patients receiving peritoneal dialysis treatment at home. Futou Bao has a series of auxiliary functions to complement this main function module.

#### Intelligent Data Upload Function Module

**Figure 4:**
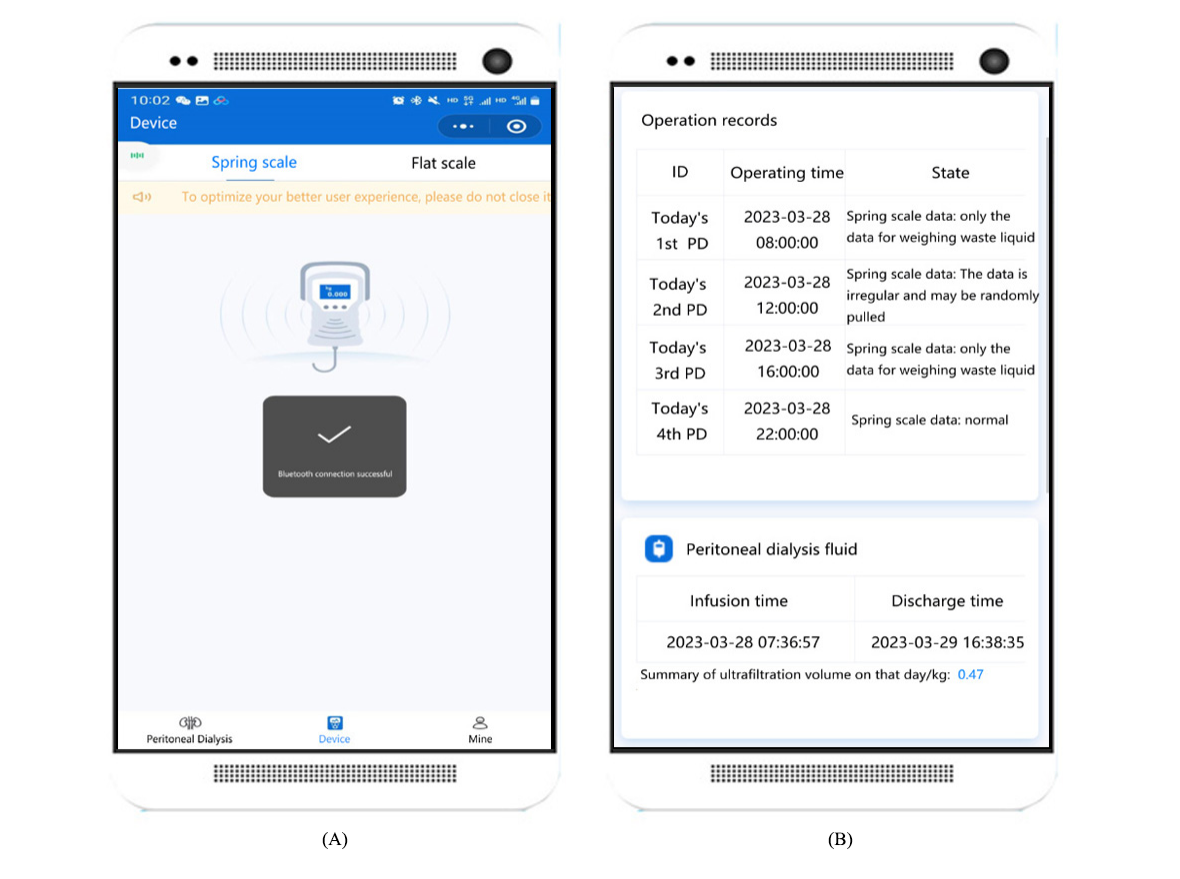
User interface of Futou Bao. (A) Uploading data through Bluetooth. (B) Peritoneal dialysis operation records. PD: peritoneal dialysis.

In this module, patients’ data during the treatment can be uploaded to the system in real time, including blood pressure, urine volume, weight, and peritoneal dialysis solution weight at different time points during peritoneal dialysis. Data can be uploaded at patients’ homes in the following 2 ways: by the patients filling in the interface of the module and by the devices automatically uploading through Bluetooth. The detailed data uploading process of the patients during peritoneal dialysis treatment is as follows (Figure 4):

1. Patients measure their own blood pressure and upload it through the module interface, including systolic and diastolic blood pressure, on the day of peritoneal dialysis.
2. Patients need to measure the urine volume per urination according to the training method and calculate the urine volume on the day of receiving peritoneal dialysis treatment and upload the data through the module interface.
3. After peritoneal dialysis, patients need to weigh themselves on the Bluetooth-enabled weighing scale, after which the weight data can be uploaded directly to the system through Bluetooth (Figure 4).
4. For the peritoneal dialysis solution weight, Futou Bao adopts the method of intelligent data collection in the whole process of peritoneal dialysis treatment. Patients need to place the peritoneal dialysis solution on a Bluetooth-enabled spring scale or electronic flat scale before the start of peritoneal dialysis treatment, and the device can upload the peritoneal dialysis solution weight data through Bluetooth in real time (Figure 4).

After uploading peritoneal dialysis data, patients can also check whether the peritoneal dialysis operation is correct or not in this module, including abnormal problems automatically identified by the system and manually marked by doctors so that patients can improve their peritoneal dialysis operation at home (Figure 4).

#### Auxiliary Function Module

There are 2 auxiliary functions to support the normal operation of the data upload function, as follows.

1. Password change and retrieve function: newly enrolled patients will have a default password, but patients can change the password according to personal needs. If patients forget the password, they can request password retrieval.
2. Bluetooth device connection management function: The patient can see the connection status of the Bluetooth-enabled scales on Futou Bao. When the patients encounter problems when connecting the Bluetooth device, the system will prompt the corresponding reason such as Bluetooth not enabled or connection timeout to help the patient solve the connection problem.

### Patients’ Data Management Platform for Doctors

**Figure 5:**
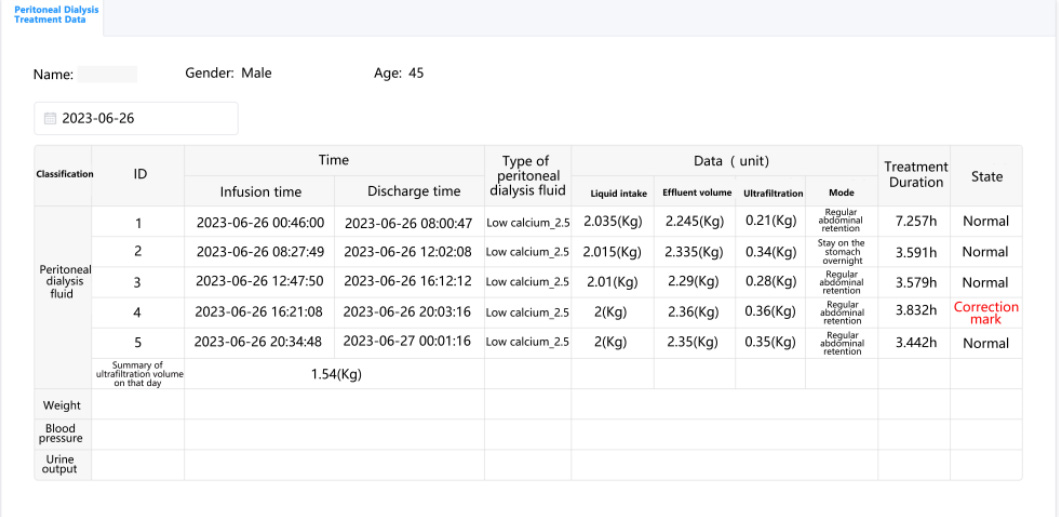
Management of patients’ daily peritoneal dialysis records, including real-time data of peritoneal dialysis fluid, weight, blood pressure, and urine output.

**Figure 6:**
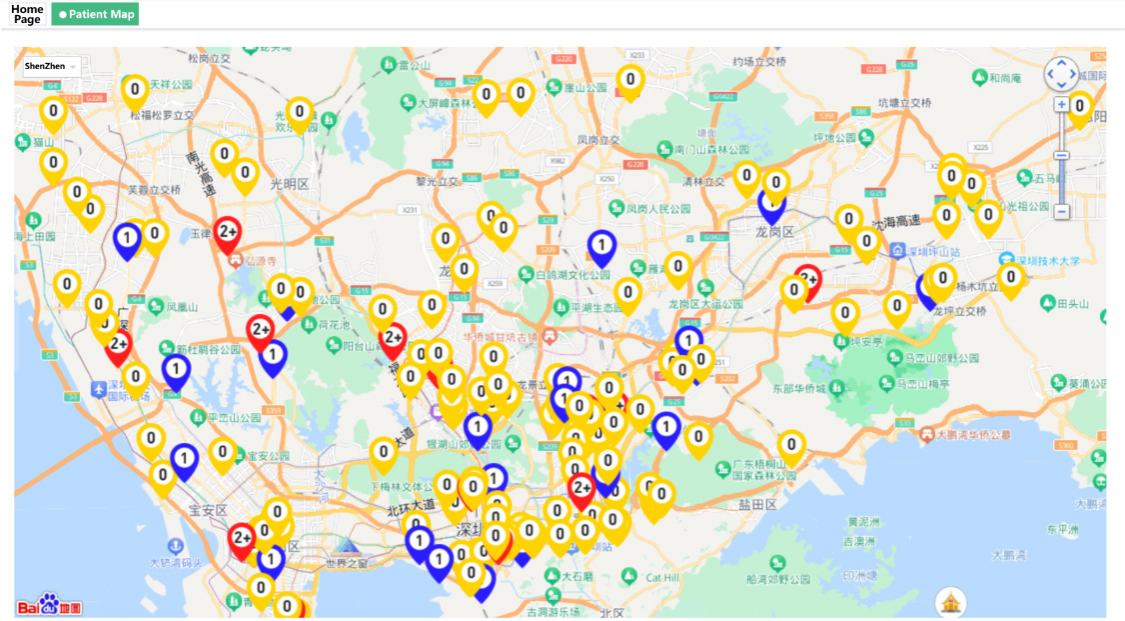
Map of patients.

**Figure 7:**
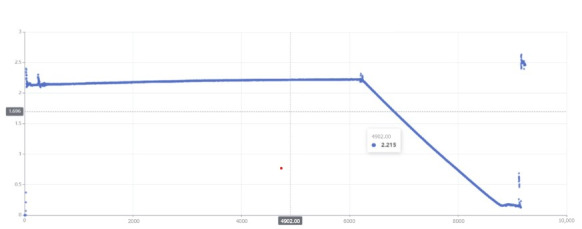
Scatterplot of the peritoneal dialysis solution weight during the treatment.

**Figure 8:**
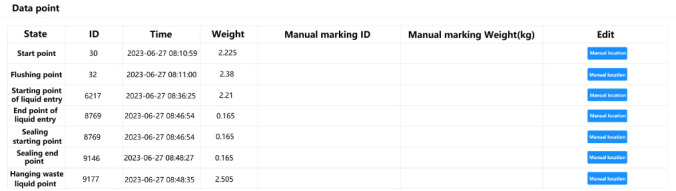
Peritoneal dialysis solution weight data at each treatment time point.

**Figure 9:**
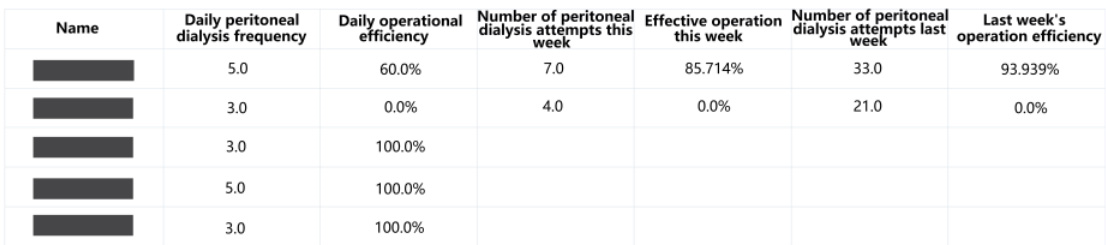
Statistics of daily and weekly peritoneal dialysis frequency and proportion of effective operations.

The management platform for doctors contains 4 function modules: patient management module, data visualization and marking module, data statistics module, and system management module (Figures 5-9).

#### Patient Management Module

There are 3 main functions in this module. The first function is that doctors can view and manage the patients’ basic information, including age, sex, date of first dialysis, and time duration of peritoneal dialysis treatment. The second function is the management of patients’ daily peritoneal dialysis records, in which doctors can query and view patients’ specific treatment records according to the treatment date (Figure 5). Based on the treatment records, doctors can know the regularity and operation of patients’ peritoneal dialysis at home. The third function in this module can provide a map of the patients’ addresses, which can help doctors to identify and help patients with operational problems during peritoneal dialysis in a timely manner (Figure 6).

#### Peritoneal Dialysis Data Visualization and Marking Module

In this module, the data visualization function can generate scatterplots of peritoneal dialysis solution weights based on the data uploaded by the spring scale or flat scale during the treatment (Figure 7). In addition to the scatterplots, doctors can also view the peritoneal dialysis solution weight data of each treatment time point, including liquid inlet starting point, liquid inlet end point, tube sealing starting point, tube sealing end point, and waste liquid hanging point (Figure 8). By viewing patient data and charts, doctors can observe patients’ peritoneal dialysis operation habits, manually mark patients’ abnormal operations and causes, and display corresponding prompts to patients through Futou Bao to correct possible wrong operations. Common marks for abnormal data include forgetting to weigh the waste liquid, only recording liquid output phase data, lack of liquid output phase data, and irregular data (which may be caused by randomly pulling the spring scale).

#### Data Statistics Module

The data statistics module consists of 4 main functions: follow-up statistics, effective operation statistics, operation time statistics of each treatment point, and comparative statistics of treatment data in recent days. First, follow-up statistics can check the number of patients’ follow-up visits, the number of new Futou Bao users, and the number of withdrawals in each year. Second, effective operation statistics function can be used to check the number of peritoneal dialysis treatments and effective operations on a given date. The effective operation rate is calculated to evaluate the overall situation of operational capability and correctness. This function makes it convenient for doctors to correct operational errors for patients (Figure 9). Third, the operation time statistics of each treatment point can be effectively viewed together with the above function. The total operation time, hand hygiene time, infusion time, and tube sealing time of the patients can be viewed. Fourth, comparative statistics of treatment data in recent days function can provide doctors with recent days’ important process data during peritoneal dialysis and mark the trends of data (ie, rising or declining), which can make it easier for doctors to know the effect of peritoneal dialysis treatment.

#### System Management Module

The system provides permission control menus based on the role-based access control method. The super administrator assigns management rights to different roles among medical staff. This process can fully ensure the meticulous management of patients by medical staff and protect the privacy of patients.

### Prediction Model of Heart Failure

#### General Information of the Study Participants

**Figure 10:**
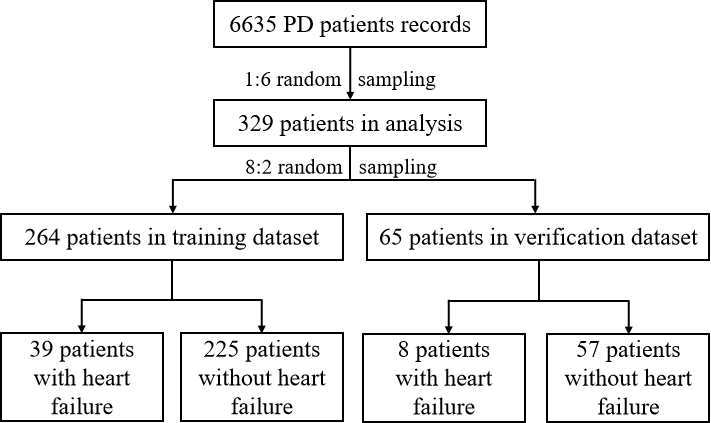
Data processing flowchart. PD: peritoneal dialysis.

During the time period of this study, records of 6635 patients who underwent peritoneal dialysis treatment were uploaded in Futou Bao with 0.71% (47/6635) showing heart failure. Based on the 1:6 random sampling, treatment data of 329 patients were included in the modeling analysis, that is, 47 (14.3%) patients with heart failure and 282 (85.7%) patients without heart failure. Table 1 shows the detailed characteristics of the included patients. Most of them were females (228/329, 69.3%), with a median age of 46.55 years and a median peritoneal dialysis time duration of 0.94 years. According to the ratio of 8:2, the study dataset was randomly divided into the training dataset and the verification dataset, which included 264 patients and 65 patients, respectively (Figure 10).

**Table 1:** Basic characteristics of the patients using Futou Bao.

Characteristics			All patients (N=6635)	Patients included in the analysis		
				Total (n=329)	Training set (n=264)	Validation set (n=65)
**Sex, n (%)**						
	Male		2578 (38.85)	101 (30.70)	79 (29.92)	22 (33.85)
	Female		4057 (61.15)	228 (69.30)	185 (70.08)	43 (66.15)
Age (years), median (IQR)			46.90 (44.68, 50.59)	46.55 (43.96, 51.19)	46.48 (43.94, 50.82)	46.91 (44.04, 52.68)
Time duration of peritoneal dialysis (years), median (IQR)			1.00 (0.53, 1.64)	0.94 (0.49, 1.78)	0.91 (0.46, 1.77)	1.08 (0.53, 1.87)
**Weight, n (%)**						
	**Based on 95% CI**					
		Normal	6115 (92.16)	299 (90.88)	239 (90.53)	60 (92.31)
		Abnormal	520 (7.84)	30 (9.12)	25 (9.47)	5 (7.69)
	**Based on 99% CI**					
		Normal	6458 (97.33)	320 (97.26)	257 (97.35)	63 (96.92)
		Abnormal	177 (2.67)	9 (2.74)	7 (2.65)	2 (3.08)
**Urine volume, n (%)**						
	**Based on 95% CI**					
		Normal	6089 (91.77)	298 (90.58)	241 (91.29)	57 (87.69)
		Abnormal	546 (8.23)	31 (9.42)	23 (8.71)	8 (12.31)
	**Based on 99% CI**					
		Normal	6384 (96.22)	313 (95.14)	252 (95.45)	61 (93.85)
		Abnormal	251 (3.78)	16 (4.86)	12 (4.55)	4 (6.15)
**Systolic blood pressure, n (%)**						
	**Based on 95% CI**					
		Normal	6189 (93.28)	299 (90.88)	239 (90.53)	60 (92.31)
		Abnormal	446 (6.72)	30 (9.12)	25 (9.47)	5 (7.69)
	**Based on 99% CI**					
		Normal	6458 (97.33)	314 (95.44)	251 (95.08)	63 (96.92)
		Abnormal	177 (2.67)	15 (4.56)	13 (4.92)	2 (3.08)
**Diastolic blood pressure, n (%)**						
	**Based on 95% CI**					
		Normal	6326 (95.34)	313 (95.14)	251 (95.08)	62 (95.38)
		Abnormal	309 (4.66)	16 (4.86)	13 (4.92)	3 (4.62)
	**Based on 99% CI**					
		Normal	6515 (98.19)	319 (96.96)	256 (96.97)	63 (96.92)
		Abnormal	120 (1.81)	10 (3.04)	8 (3.03)	2 (3.08)
**Pulse pressure difference, n (%)**						
	**Based on 95% CI**					
		Normal	6232 (93.93)	299 (90.88)	238 (90.15)	61 (93.85)
		Abnormal	403 (6.07)	30 (9.12)	26 (9.85)	4 (6.15)
	**Based on 99% CI**					
		Normal	6478 (97.63)	316 (96.05)	254 (96.21)	62 (95.38)
		Abnormal	157 (2.37)	13 (3.95)	10 (3.79)	3 (4.62)
**Heart failure, n (%)**						
	Yes		47 (0.71)	47 (14.29)	39 (14.77)	8 (12.31)
	No		6588 (99.29)	282 (85.71)	225 (85.23)	57 (87.69)

#### Optimal Prediction Model Selection

**Figure 11:**
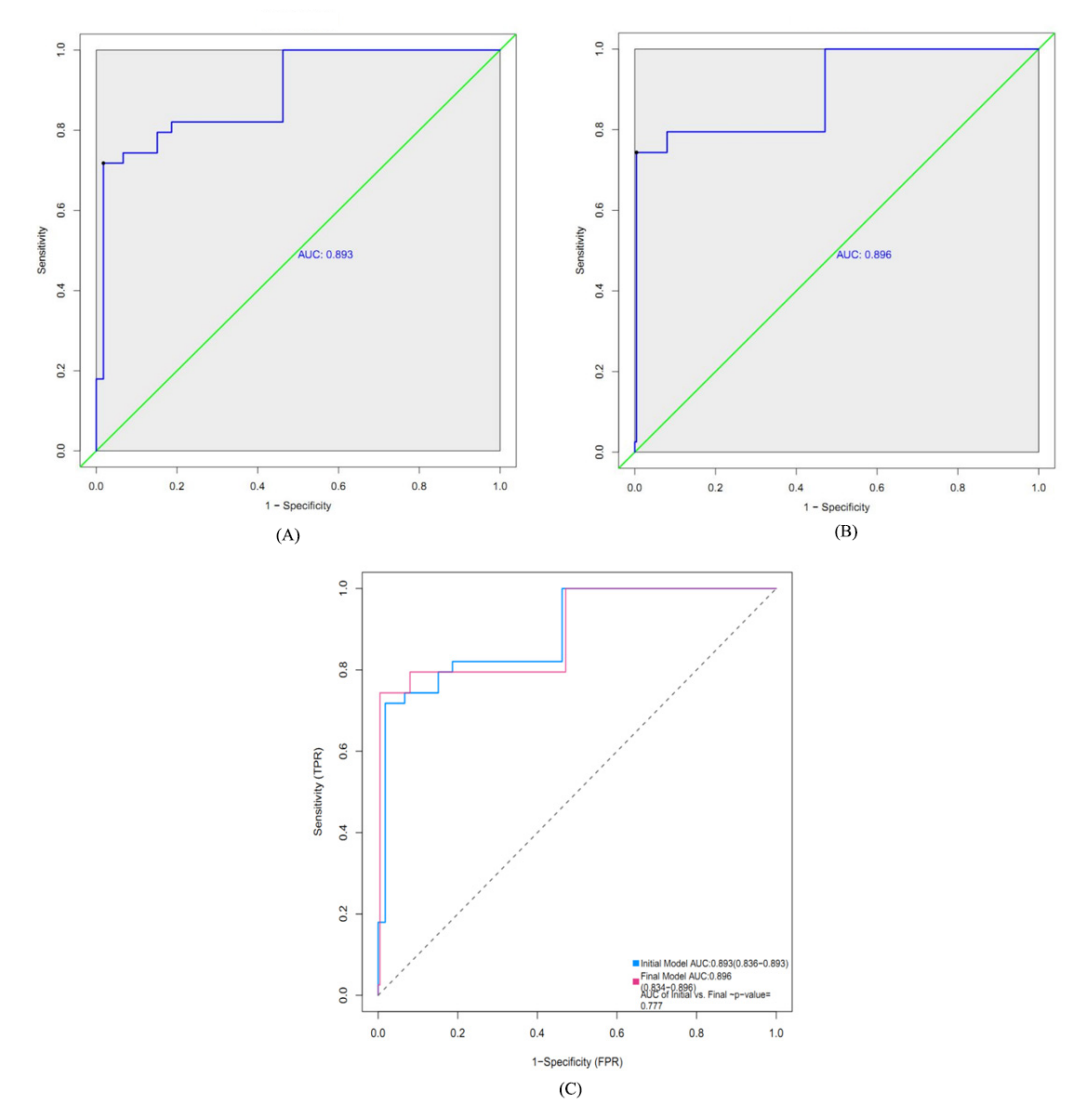
Receiver operating characteristic curves of 2 heart failure prediction models in the training dataset. (A) Initial model (model 1). (B) Final model (model 4). (C) Model comparison. AUC: area under the curve; FPR: false positive rate; TPR: true positive rate.

Four prediction models based on logistic regression were constructed using the training dataset (Tables 2-3). The AIC values of the 4 models were 147.23, 138.62, 145.18, and 136.50, respectively. The AUC value of model 4 (the lowest AIC value) was higher than that of model 1 (the highest AIC value) (AUC_Model 4_ vs AUC_Model 1_ : 0.896 vs 0.893); however, the difference was not statistically significant (*P*=.78) (Figure 11). Based on the above comparison, model 4 was considered as the optimal prediction model. Sex, age, and diastolic blood pressure were included in model 4. Females were less likely to experience heart failure than males (adjusted odds ratio [AOR] 0.06, 95% CI 0.01-0.22), while older age (AOR 1.19, 95% CI 1.13-1.25) and abnormal diastolic blood pressure during treatment (AOR 38.79, 95% CI 5.08-291.63) were the risk factors of heart failure (Table 3).

**Table 2:** Impact factors of heart failure in patients receiving peritoneal dialysis treatment (abnormal value based on 95% CI).

Characteristics		Univariable model			Multivariable model 1			Multivariable model 2	
		Odds ratio (95% CI)	*P* value	AOR^a^ (95% CI)		*P* value	AOR (95% CI)		*P* value
**Sex**									
	Male	1.00	N/A^b^	1.00		N/A	N/A		N/A
	Female	0.10 (0.02-0.35)	.002	0.05 (0.01-0.22)		<.001	0.05 (0.01-0.26)		<.001
Age		1.17 (1.12-1.23)	<.001	1.18 (1.11-1.28)		<.001	1.17 (1.12-1.23)		<.001
Time duration of peritoneal dialysis		2.73 (1.82-4.32)	<.001	0.92 (0.42-1.80)		.83	N/A		N/A
**Weight**									
	Normal	1.00	N/A	1.00		N/A	N/A		N/A
	Abnormal	0.48 (0.07-1.70)	.33	1.24 (0.15-6.22)		.81	N/A		N/A
**Urine volume**									
	Normal	1.00	N/A	1.00		N/A	N/A		N/A
	Abnormal	3.61 (1.36-9.06)	.007	1.54 (0.31-7.84)		.59	N/A		N/A
**Systolic blood pressure**									
	Normal	1.00	N/A	1.00		N/A	N/A		N/A
	Abnormal	4.83 (1.94-11.68)	.001	4.49 (0.62-45.50)		.17	8.02 (2.09-30.77)		.002
**Diastolic blood pressure**									
	Normal	1.00	N/A	1.00		N/A	N/A		N/A
	Abnormal	2.74 (0.71-8.93)	.11	2.31 (0.22-17.93)		.44	N/A		N/A
**Pulse pressure difference**									
	Normal	1.00	N/A	1.00		N/A	N/A		N/A
	Abnormal	2.37 (0.87-5.59)	.07	1.05 (0.11-6.83)		.96	N/A		N/A

^a^AOR: adjusted odds ratio.

^b^N/A: not applicable.

**Table 3:** Impact factors of heart failure in patients receiving peritoneal dialysis treatment (abnormal value based on 99% CI).

Characteristics			Univariable model					Multivariable model 3					Multivariable model 4		
			Odds ratio (95% CI)		*P* value		AOR^a^ (95% CI)			*P* value		AOR (95% CI)			*P* value
**Sex**															
	Male	1.00		N/A^b^		1.00			N/A		1.00			N/A	
	Female	0.10 (0.02-0.35)		.002		0.04 (0.01-0.22)			.001		0.06 (0.01-0.22)			<.001	
Age			1.17 (1.12-1.23)		<.001		1.20 (1.12-1.30)			<.001		1.19 (1.13-1.25)			<.001
Time duration of peritoneal dialysis			2.73 (1.82-4.32)		<.001		0.95 (0.41-1.89)			.89		N/A			N/A
**Weight**															
	Normal	1.00		N/A		1.00			N/A		N/A			N/A	
	Abnormal	2.38 (0.33-11.49)		.31		4.34 (0.23-44.51)			.25		N/A			N/A	
**Urine volume**															
	Normal	1.00		N/A		1.00			N/A		N/A			N/A	
	Abnormal	3.10 (0.79-10.41)		.08		1.19 (0.11-12.44)			.88		N/A			N/A	
**Systolic blood pressure**															
	Normal	1.00		N/A		1.00			N/A		N/A			N/A	
	Abnormal	3.99 (1.15-12.69)		.02		1.17 (0.04-18.27)			.91		N/A			N/A	
**Diastolic blood pressure**															
	Normal	1.00		N/A		1.00			N/A		1.00			N/A	
	Abnormal	6.31 (1.44-27.83)		.01		9.87 (0.07-290.03)			.41		38.79 (5.08-291.63)			<.001	
**Pulse pressure difference**															
	Normal	1.00		N/A		1.00			N/A		N/A			N/A	
	Abnormal	4.17 (1.02-15.35)		.03		1.35 (0.01-74.38)			.90		N/A			N/A	

^a^AOR: adjusted odds ratio.

^b^N/A: not applicable.

#### Validation of the Optimal Prediction Model

**Figure 12:**
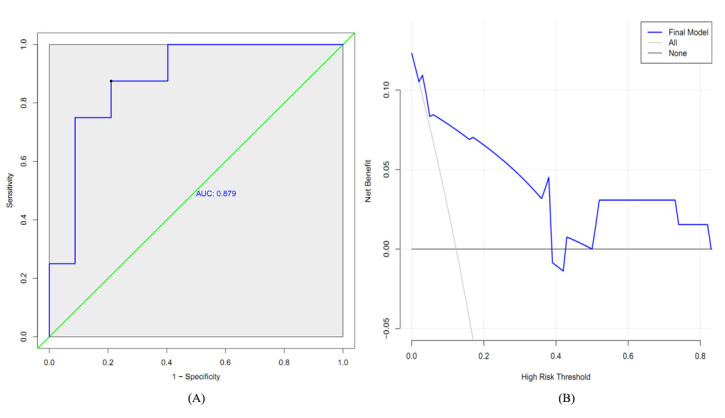
Performance of the final model in the testing dataset. (A) Receiver operating characteristic curve. (B) Decision curve. AUC: area under the curve.

Based on the validation dataset, the sensitivity, specificity, accuracy, and Youden index of the optimal model were 0.75, 0.91, 0.89, and 0.66, respectively. Figure 12A shows the ROC curve of the model, of which the AUC value was 0.879 (95% CI 0.772-0.986). The decision curve also showed the acceptable performance of the model (Figure 12B).

#### Sensitivity Analysis

In the sensitivity analysis, after changing the ratio of patients with and without heart failure (1:4 and 1:10), the analysis showed stable results. The variables included in the optimal model were constant, including sex, age, and diastolic blood pressure during treatment (Tables S1-S2 of Multimedia Appendix 1). In validation, the models also showed good performance. The AUC values were 0.877 (95% CI 0.774-0.980) in the 1:4 ratio dataset and 0.913 (95% CI 0.825-0.999) in the 1:10 ratio datasets, respectively (Figures S1-S2 in Multimedia Appendix 1). The Youden indexes were 0.76 and 0.68 in the 2 datasets, respectively.

## Discussion

### Principal Findings

In this study, a practical and user-friendly mHealth tool named Futou Bao and its supporting management system were developed for patients with ESRD who managed their peritoneal dialysis treatment at home in China. With its comprehensive functions that provided intelligent data upload, visualization, management, and doctor-patient interaction, the mHealth tool and system could effectively solve the difficulties in managing peritoneal dialysis treatment at home, increase treatment effectiveness, and minimize complication risk. Compared with the CKD-PD (chronic kidney disease-peritoneal dialysis) app [12] developed by researchers in the Khon Kaen University, Thailand, in the same period as our study, Futou Bao had several additional advantages. First, automatic data upload via Bluetooth could reduce data recording errors. Second, the recording of peritoneal dialysis solution weight data was more detailed, which included the weight data at each time point during the whole treatment process, and it could generate data visualization plots timely. Third, Futou Bao could provide better interactions between doctors and patients. The operation problems marked by doctors can be notified to the patients through the platform in a timely manner to help patients improve the peritoneal dialysis operation quality. Fourth, the Futou Bao system was able to locate patients’ addresses to detect serious health risks and provide timely medical assistance. In addition, a previous study conducted among Thai patients using the CKD-PD app mainly focused on the optimization of the app’s functionality and usefulness with only 10 participants involved [12]. By contrast, in this study, a larger number of patients were included to explore a better way for assisting clinical decision-making with the help of constructing prediction models based on the usage records of Futou Bao. We found that most of the patients with ESRD using Futou Bao, as an eHealth management and promoting tool, in our study were females, which was different from the real-world clinical experiences, wherein most patients treated for ESRD with any form of dialysis were males. However, in the process of recruitment, we only relied on patients’ voluntary participation in the study and did not induce them in any form or select any of them based on their sex. This interesting phenomenon can be explained by the higher acceptance of the eHealth approaches among females. A study focusing on drug use treatment in China using a mobile app also showed that female users were more willing to receive health care messages and communicate with health workers through the mobile app and were more likely to have positive health changes than male users, which could support our finding [13].

Given the importance of early detection of heart failure in patients receiving home-based peritoneal dialysis, it is of great value to make full use of the data recorded in the process of daily peritoneal dialysis treatment to perform real-time prediction and for early warning of heart failure risk. However, the variables recorded during the daily home-based peritoneal dialysis were limited to some extent. In previous studies, the prediction models of heart diseases among patients with CKD and ESRD mainly relied on clinical test indicators and biomarkers that could not be tested at home. Kessler et al [14] built a risk prediction model of cardiovascular events for patients with ESRD based on the FOSIDIAL trial and found that C-reactive protein and left ventricular mass index were important prognostic factors of cardiovascular events. Weekley and Peralta [15] indicated that combining creatinine, cystatin C, and albuminuria could improve the detection and risk stratification for heart failure and other cardiovascular events. Other biomarkers, including estimated glomerular filtration rate, urine albumin-to-creatinine ratio, and β_2_-microglobulin, were also proved to be associated with heart failure in patients with CKD [16-20]. Based on evidences from the Chronic Renal Insufficiency Cohort study, a Pooled Cohort Equations to Prevent Heart Failure in the CKD population was constructed to predict incident heart failure hospitalizations [21]. A series of clinical factors were included in this model, such as blood pressure, fasting glucose, total cholesterol, high-density lipoprotein cholesterol, QRS duration, estimated glomerular filtration rate, and albuminuria [21]. A recent research based on the data of a Chronic Renal Insufficiency Cohort study indicated that adding N-terminal brain natriuretic peptide and high sensitivity troponin-T to the prediction model could improve the discrimination of heart failure [22].

Considering the unavailability of laboratory test results in daily home-based peritoneal dialysis and the lack of heart failure prediction model in patients receiving peritoneal dialysis treatment, our study innovatively establishes a practical prediction model of heart failure in patients undergoing peritoneal dialysis based on the monitoring data of daily peritoneal dialysis treatment at home. The factors in the prediction model included sex, age, time duration of peritoneal dialysis, weight, urine volume, and blood pressure, which were accessible and real-time data monitored and recorded by the Futou Bao. In addition, the effectiveness of the prediction model based on routine monitoring variables was acceptable. In the model validation, the accuracy of the prediction model could reach 89%, with sensitivity of 75% and higher specificity of 91%. Therefore, the mHealth tools and the predictive model developed in this study enabled monitoring and early warning of heart failure risk, which has formed a new paradigm for the management of patients receiving home-based peritoneal dialysis treatment. From another perspective, during the 5-year follow-up, the proportion of patients experiencing heart failure in our study was less than 1%, which was much lower than that reported in previous studies [7-10]. This result could indicate that our management paradigm was practical and effective.

### Limitations

There were several limitations in this study. First, the number of patients with heart failure during the observation period of this study was small, which resulted in a relatively small number of patients in the dataset for the prediction model study. Volunteer bias may enhance the management effectiveness of the system. In future studies, we will conduct randomized controlled trials to better rule out confounding factors and reduce the occurrence of bias. Second, the mHealth tools were only used for daily data collection and monitoring, and the exclusion criteria were relatively strict that patients with previous heart failure or coronary heart disease history were not included in this study. To better expand the coverage of patients included and managed in the future, more variables in their medical history should be added to better predict the risk of heart failure. Third, the mHealth system was only used in the scenario of large cities represented by Shenzhen. In future studies, the regional scope of the system should be further expanded. In addition, the sex and age composition of the users was different from that in clinical practice experiences. In the future, the performance of Futou Bao should be promoted in the following 3 ways: (1) further investigation is needed to explore the obstacles in male users’ acceptance of Futou Bao, and the functional design should consider the needs of both male and female users; (2) the improvement of Futou Bao should enhance the usage experience of older adults to help them use the tool more conveniently; and (3) on the basis of ensuring patient privacy, family caregivers can be authorized to use the patient platform so that more older adults who do not have the ability to use smartphones can benefit from using this home-based system.

### Conclusions

This is a pioneering study that describes the development of an intelligent home-based peritoneal dialysis management and monitoring system based on mHealth technology in China. This system consists of a WeChat Mini Program named Futou Bao for patients, which includes an intelligent data upload function module and an auxiliary function module; and a patient data management platform for doctors, which includes 4 function modules: patient management, data visualization and marking, data statistics, and system management. Based on the daily peritoneal dialysis treatment data recorded in the system, a real-time prediction model of heart failure in patients receiving peritoneal dialysis treatment was built and verified. This accuracy of the prediction model was accepted, with the AUC value of 0.879 (95% CI 0.772-0.986). The new home-based peritoneal dialysis management paradigm that we developed enabled real-time monitoring and early warning of heart failure risk. In the future, it is essential to evaluate the effectiveness of mHealth tools through multicenter studies, which should include regions with different economic levels and medical and health service levels to improve the universality of the research results.

## Appendix

Multimedia Appendix 1 Supplementary data.

## Abbreviations

AIC: Akaike’s information criterion
AOR: adjusted odds ratio
AUC: area under the curve
CAPD: continuous ambulatory peritoneal dialysis
CKD-PD: chronic kidney disease-peritoneal dialysis
ESRD: end-stage renal disease
mHealth: mobile health

## Data Availability

The datasets in this study are available from the corresponding author upon reasonable request.
