# Supplementary material for: Daily Treatment Monitoring for Patients Receiving Home-Based Peritoneal Dialysis and Prediction of Heart Failure Risk: mHealth Tool Development and Modeling Study
Source: JMIR Form Res. 2025 Mar 3;9:e56254. doi: 10.2196/56254 (PMC11914845; doi:10.2196/56254)
Supplement: Multimedia Appendix 1 [file formative_v9i1e56254_app1.docx]

**Multimedia Appendix 1**

**Table S1. Impact factors of heart failure in patients receiving peritoneal dialysis treatment**

**(Number of patients with heart failure vs Number of patients without heart failure = 1:4)**

| **Characteristics** | | **Abnormal value based on 95%CI** | | | | | |  | **Abnormal value based on 99%CI** | | | | | |
| --- | --- | --- | --- | --- | --- | --- | --- | --- | --- | --- | --- | --- | --- | --- |
|  |  | **Univariable model** | | **Mutivariable model** | | | |  | **Univariable model** | | **Mutivariable model** | | | |
|  |  |  |  | **Model 1** | | **Model 2** | |  |  |  | **Model 3** | | **Model 4** | |
|  |  | OR (95%CI) | P value | AOR (95%CI) | P value | AOR (95%CI) | P value |  | OR (95%CI) | P value | AOR (95%CI) | P value | AOR (95%CI) | P value |
| Sex | |  |  |  |  |  |  |  |  |  |  |  |  |  |
|  | male | 1.00 | — | 1.00 | — | — | — |  | 1.00 | — | 1.00 | — | 1.00 | — |
|  | female | 0.05 (0.01-0.26) | 0.005 | 0.07 (0.01-0.24) | <0.001 | 0.07 (0.01-0.30) | <0.001 |  | 0.05 (0.01-0.26) | 0.005 | 0.06 (0.01-0.25) | <0.001 | 0.07 (0.02-0.31) | <0.001 |
| Age | | 1.16 (1.11-1.22) | <0.001 | 1.16 (1.10-1.23) | <0.001 | 1.16 (1.11-1.21) | <0.001 |  | 1.16 (1.11-1.22) | <0.001 | 1.17 (1.10-0.24) | <0.001 | 1.17 (1.12-1.23) | <0.001 |
| Time duration of PD | | 3.91 (2.40-6.73) | <0.001 | 1.07 (0.57-1.87) | 0.810 | — | — |  | 3.91 (2.40-6.73) | <0.001 | 1.06 (0.57-1.88) | 0.832 | — | — |
| Weight | |  |  |  |  |  |  |  |  |  |  |  |  |  |
|  | Normal | 1.00 | — | 1.00 | — | — | — |  | 1.00 | — | 1.00 | — | — | — |
|  | Abnormal | 0.78 (0.12-3.12) | 0.752 | 0.78 (0.10-3.74) | 0.781 | — | — |  | 2.00 (0.09-21.43) | 0.576 | 0.89 (0.07-7.02) | 0.920 | — | — |
| Urine volume | |  |  |  |  |  |  |  |  |  |  |  |  |  |
|  | Normal | 1.00 | — | 1.00 | — | — | — |  | 1.00 | — | 1.00 | — | — | — |
|  | Abnormal | 5.51 (1.95-15.86) | 0.001 | 1.77 (0.46-6.51) | 0.392 | — | — |  | 4.29 (0.97-19.00) | 0.047 | 2.71 (0.26-25.54) | 0.391 | — | — |
| Systolic blood pressure | | |  |  |  |  |  |  |  |  |  |  |  |  |
|  | Normal | 1.00 | — | 1.00 | — | — | — |  | 1.00 | — | 1.00 | — | — | — |
|  | Abnormal | 3.92 (1.46-10.35) | 0.006 | 3.91 (0.97-15.81) | 0.05 | 5.10 (1.84-14.11) | 0.002 |  | 5.53 (1.39-23.42) | 0.014 | 2.16 (0.08-24.76) | 0.575 | — | — |
| Diastolic blood pressure | | |  |  |  |  |  |  |  |  |  |  |  |  |
|  | Normal | 1.00 | — | 1.00 | — | — | — |  | 1.00 | — | 1.00 | — | 1.00 | — |
|  | Abnormal | 2.82 (0.69-10.44) | 0.123 | 2.99 (0.45-16.07) | 0.223 | — | — |  | 8.71 (1.63-64.67) | 0.015 | 9.67 (0.23-642.26) | 0.254 | 27.41 (4.45-168.87) | <0.001 |
| Pulse pressure difference | | |  |  |  |  |  |  |  |  |  |  |  |  |
|  | Normal | 1.00 | — | 1.00 | — | — | — |  | 1.00 | — | 1.00 | — | — | — |
|  | Abnormal | 1.98 (0.65-5.42) | 0.200 | 0.74 (0.14-3.42) | 0.708 | — | — |  | 3.41 (0.81-13.56) | 0.078 | 0.63 (0.03-6.42) | 0.722 | — | — |

Note: AOR: adjusted odds ratio; CI: confidence interval; PD: peritoneal dialysis.

**Figure S1. Performance of final model in testing dataset**

**(Number of patients with heart failure vs Number of patients without heart failure = 1:4)**


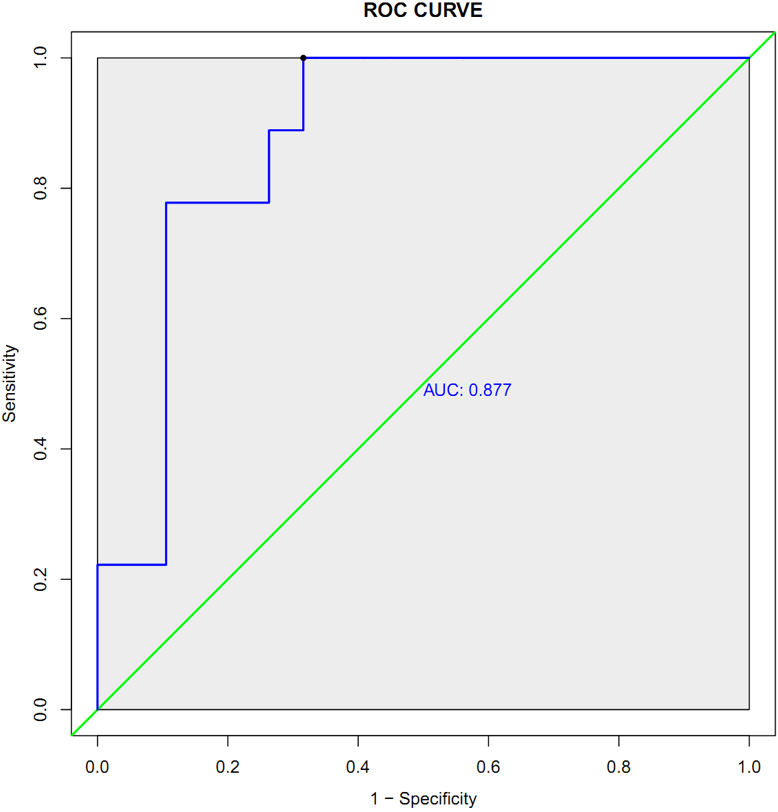

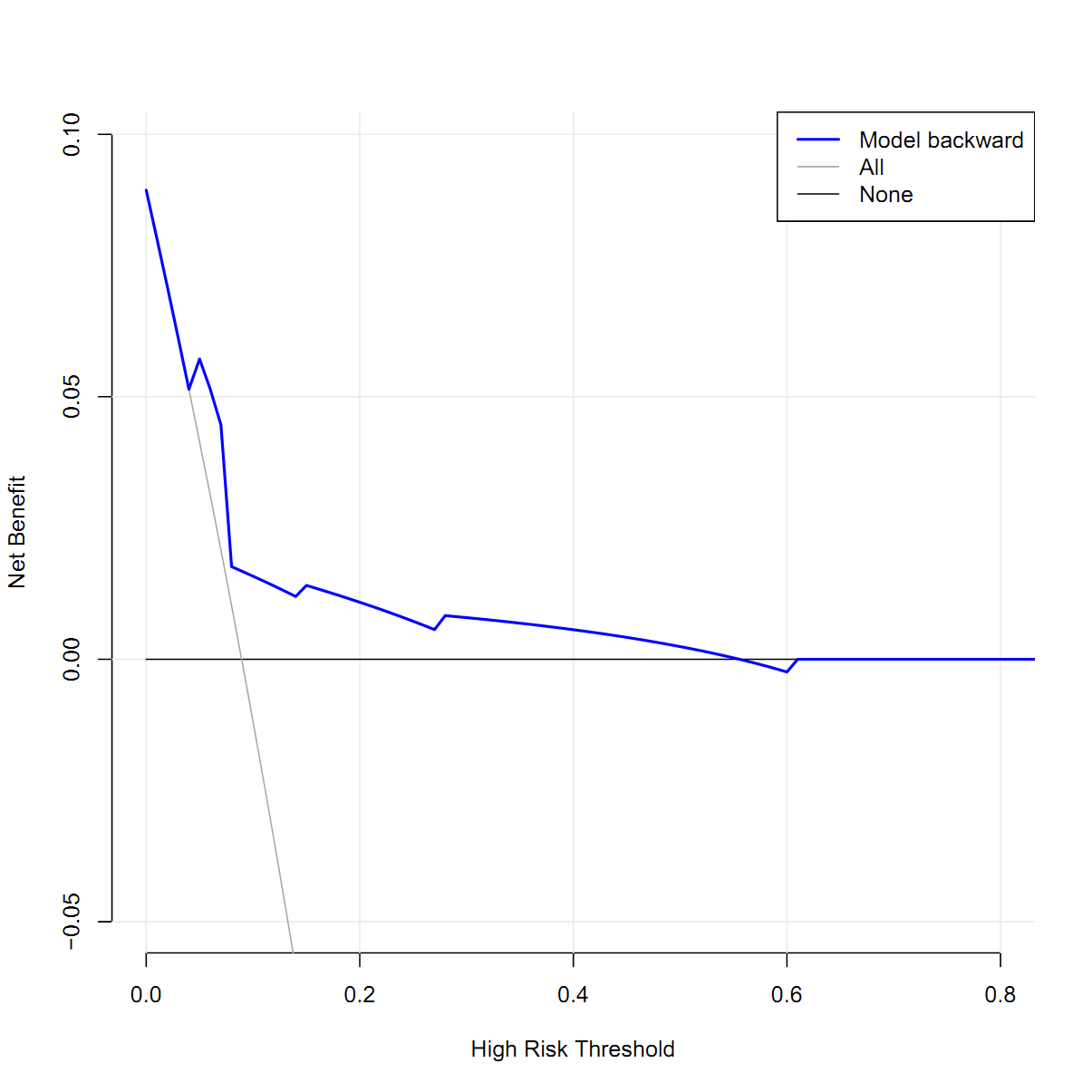


(A) ROC curve (B) Decision curve

**Table S2. Impact factors of heart failure in patients receiving peritoneal dialysis treatment**

**(Number of patients with heart failure vs Number of patients without heart failure = 1:10)**

| **Characteristics** | | **Abnormal value based on 95%CI** | | | | | |  | **Abnormal value based on 99%CI** | | | | | |
| --- | --- | --- | --- | --- | --- | --- | --- | --- | --- | --- | --- | --- | --- | --- |
|  |  | **Univariable model** | | **Mutivariable model** | | | |  | **Univariable model** | | **Mutivariable model** | | | |
|  |  |  |  | **Model 1** | | **Model 2** | |  |  |  | **Model 3** | | **Model 4** | |
|  |  | OR (95%CI) | P value | AOR (95%CI) | P value | AOR (95%CI) | P value |  | OR (95%CI) | P value | AOR (95%CI) | P value | AOR (95%CI) | P value |
| Sex | |  |  |  |  |  |  |  |  |  |  |  |  |  |
|  | male | 1.00 | — | 1.00 | — | — | — |  | 1.00 | — | 1.00 | — | 1.00 | — |
|  | female | 0.11 (0.02-0.35) | 0.002 | 0.07 (0.01-0.24) | <0.001 | 0.07 (0.01-0.30) | <0.001 |  | 0.11 (0.02-0.35) | 0.002 | 0.06 (0.01-0.25) | <0.001 | 0.07 (0.02-0.31) | <0.001 |
| Age | | 1.16 (1.11-1.21) | <0.001 | 1.16 (1.10-1.23) | <0.001 | 1.16 (0.11-1.21) | <0.001 |  | 1.16 (1.11-1.21) | <0.001 | 1.17 (1.11-1.24) | <0.001 | 1.17 (1.12-1.23) | <0.001 |
| Time duration of PD | | 1.87 (1.38-2.60) | <0.001 | 1.07 (0.57-1.87) | 0.810 | — | — |  | 1.87 (1.38-2.60) | <0.001 | 1.06 (0.57-1.88) | 0.832 | — | — |
| Weight | |  |  |  |  |  |  |  |  |  |  |  |  |  |
|  | Normal | 1.00 | — | 1.00 | — | — | — |  | 1.00 | — | 1.00 | — | — | — |
|  | Abnormal | 0.71 (0.11-2.51) | 0.652 | 0.78 (0.10-3.73) | 0.781 | — | — |  | 2.34 (0.35-9.52) | 0.290 | 0.89 (0.07-7.02) | 0.920 | — | — |
| Urine volume | |  |  |  |  |  |  |  |  |  |  |  |  |  |
|  | Normal | 1.00 | — | 1.00 | — | — | — |  | 1.00 | — | 1.00 | — | — | — |
|  | Abnormal | 3.44 (1.36-7.96) | 0.006 | 1.77 (0.46-6.51) | 0.393 | — | — |  | 3.39 (0.92-10.23) | 0.042 | 2.71 (0.26-25.54) | 0.391 | — | — |
| Systolic blood pressure | | |  |  |  |  |  |  |  |  |  |  |  |  |
|  | Normal | 1.00 | — | 1.00 | — | — | — |  | 1.00 | — | 1.00 | — | — | — |
|  | Abnormal | 5.48 (2.38-12.11) | <0.001 | 3.90 (0.97-15.81) | 0.054 | 5.10 (1.84-14.11) | 0.002 |  | 5.20 (1.56-15.27) | 0.004 | 2.16 (0.08-24.76) | 0.575 | — | — |
| Diastolic blood pressure | | |  |  |  |  |  |  |  |  |  |  |  |  |
|  | Normal | 1.00 | — | 1.00 | — | — | — |  | 1.00 | — | 1.00 | — | 1.00 | — |
|  | Abnormal | 2.42 (0.67-6.94) | 0.129 | 2.99 (0.45-16.07) | 0.223 | — | — |  | 7.50 (1.84-27.58) | 0.003 | 9.67 (0.23-642.26) | 0.254 | 27.41 (4.45-168.87) | <0.001 |
| Pulse pressure difference | | |  |  |  |  |  |  |  |  |  |  |  |  |
|  | Normal | 1.00 | — | 1.00 | — | — | — |  | 1.00 | — | 1.00 | — | — | — |
|  | Abnormal | 3.03 (1.14-7.21) | 0.017 | 0.74 (0.14-3.42) | 0.708 | — | — |  | 3.69 (0.99-11.28) | 0.031 | 0.63 (0.03-6.42) | 0.722 | — | — |

Note: AOR: adjusted odds ratio; CI: confidence interval; PD: peritoneal dialysis.

**Figure S2. Performance of final model in testing dataset**

**(Number of patients with heart failure vs Number of patients without heart failure = 1:10)**


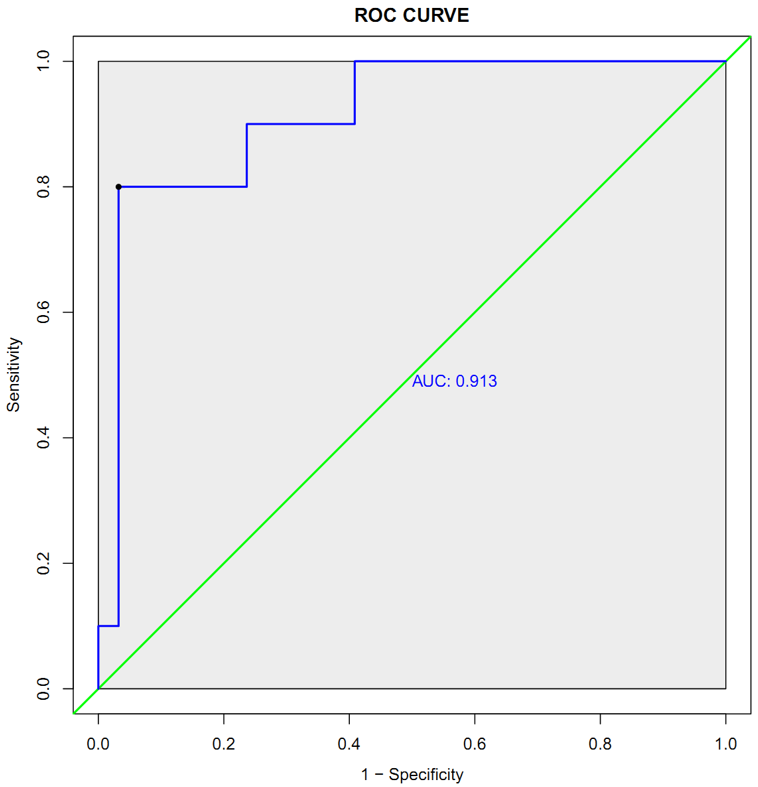

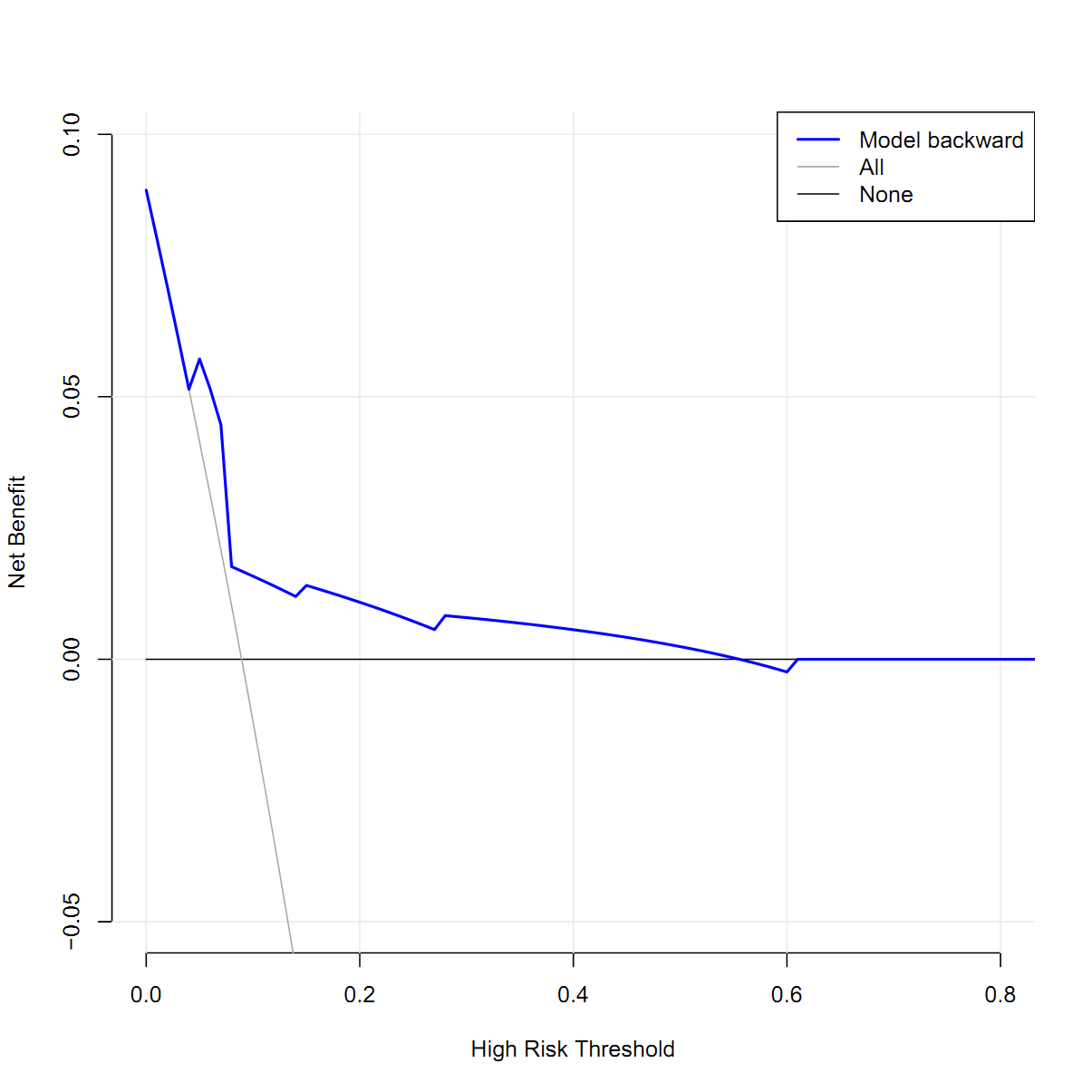


(A) ROC curve (B) Decision curve
